# Supplementary material for: Clustering assessment in weighted networks
Source: PeerJ Comput Sci. 2021 Jun 18;7:e600. doi: 10.7717/peerj-cs.600 (PMC8237321; doi:10.7717/peerj-cs.600)
Supplement: Supplemental Information 2 [file peerj-cs-07-600-s002.pdf]

SUPPLEMENTAL TABLES S1

Clustering assessment in weighted networks

Argimiro Arratia, Martí Renedo Mirambell
Department of Computer Sciences, Polytechnical University of Catalonia, Barcelona, Spain

	Louvain	leading eigen	label prop	Walktrap	spin-glass	ground truth
$\uparrow$ internal density	0.39|0.479(0.02)	0.384|0.362(0.64)	0.311|0.131(1)	0.39|0.33(0.83)	0.46|0.531(0.11)	3.90e-01
$\uparrow$ edges inside	197|42.6(1)	194|61(1)	252|640(0)	196|22.4(1)	95.7|40.9(1)	1.96e+02
$\uparrow$ av degree	11.5|5.77(1)	11.3|5.62(1)	11.7|12.8(0)	11.4|2.66(1)	8.92|5.85(1)	1.15e+01
$\uparrow$ FOMD	0.38|0.0805(1)	0.38|0.0939(1)	0.38|0.45(0)	0.38|0.0359(1)	0.25|0.084(1)	3.80e-01
$\downarrow$ expansion	0.67|3.51(0)	0.73|3.59(0)	0.53|0.16(1)	0.68|5.07(0)	1.94|3.47(0)	6.70e-01
$\downarrow$ cut ratio	0.00931|0.041(0)	0.00997|0.0444(0)	0.00783|0.0408(0)	0.00945|0.0581(0)	0.0245|0.0401(0)	9.31e-03
$\downarrow$ conductance	0.0785|0.381(0)	0.089|0.398(0)	0.0621|0.0125(1)	0.08|0.654(0)	0.159|0.375(0)	7.86e-02
$\downarrow$ norm cut	0.101|0.42(0)	0.112|0.452(0)	0.0839|0.148(0)	0.103|0.699(0)	0.195|0.412(0)	1.01e-01
$\downarrow$ max ODF	0.164|0.528(0)	0.215|0.552(0)	0.203|0.00875(1)	0.222|0.673(0)	0.266|0.521(0)	1.66e-01
$\downarrow$ average ODF	0.142|0.544(0)	0.157|0.563(0)	0.113|0.0259(1)	0.144|0.762(0)	0.272|0.536(0)	1.41e-01
$\downarrow$ flake ODF	0.72|0.996(0)	0.72|0.994(0)	0.62|0.17(1)	0.7|0.975(0)	0.9|0.995(0)	7.10e-01
$\uparrow$ clustering coef	0.312|0.011(1)	0.31|0.00904(1)	0.303|0.00563(1)	0.312|0.00402(1)	0.352|0.0109(1)	3.12e-01
$\uparrow$ modularity	0.454|0.307(1)	0.447|0.242(1)	0.411|0.000267(1)	0.453|0.082(1)	0.446|0.321(1)	4.54e-01
n_communities	4|7.8(0)	4|6.84(0.02)	3|1.01(1)	4|21.8(0)	5|8.51(0)	4.00e+00
VIdist_to_GT	0.122|4.45(0)	0.299|4.17(0)	0.361|1.86(0)	0.122|4.35(0)	0.521|4.52(0)	0.00e+00

Table 1. Values of scoring functions for the weighted SBM graph generated with $\lambda = 15$ (left), compared to those of their averages for 100 randomized samples (right), and its percentile rank (parentheses).

	Louvain	leading eigen	label prop	Walktrap	spin-glass	ground truth
$\uparrow$ internal density	1.29|1.6(0.13)	1.32|1.24(0.69)	1.07|1.17(0.4)	1.32|1.5(0.42)	1.29|1.7(0.06)	0.7574
$\uparrow$ edges inside	50.4|30.5(0.99)	47.4|37.5(0.82)	83.4|106(0.57)	51.8|41.9(0.75)	50.4|28.6(1)	103.0000
$\uparrow$ av degree	10.1|8.26(1)	9.94|7.98(1)	11.6|10.4(0.72)	10.1|8.44(0.9)	10.1|8.21(1)	12.1176
$\uparrow$ FOMD	0.412|0.29(0.99)	0.412|0.273(0.99)	0.471|0.38(0.71)	0.382|0.299(0.82)	0.412|0.288(0.98)	0.4412
$\downarrow$ expansion	1.74|2.67(0)	1.82|2.81(0)	0.971|1.99(0.0886)	1.74|2.57(0.08)	1.74|2.69(0)	0.7353
$\downarrow$ cut ratio	0.0716|0.0994(0)	0.0739|0.11(0)	0.0469|0.0994(0.0253)	0.0727|0.101(0.06)	0.0716|0.0987(0)	0.0433
$\downarrow$ conductance	0.149|0.257(0)	0.179|0.293(0.02)	0.0784|0.18(0.114)	0.148|0.273(0.05)	0.149|0.264(0)	0.0573
$\downarrow$ norm cut	0.194|0.302(0)	0.223|0.349(0)	0.119|0.25(0)	0.194|0.328(0.01)	0.194|0.307(0)	0.1038
$\downarrow$ max ODF	0.216|0.394(0)	0.246|0.396(0.04)	0.0441|0.274(0.0506)	0.211|0.382(0.06)	0.216|0.399(0.01)	0.0833
$\downarrow$ average ODF	0.183|0.389(0)	0.215|0.442(0)	0.114|0.292(0.0759)	0.185|0.391(0.01)	0.183|0.397(0)	0.0871
$\downarrow$ flake ODF	0.559|0.946(0)	0.588|0.941(0)	0.5|0.827(0.0759)	0.559|0.921(0.01)	0.559|0.95(0)	0.3824
$\uparrow$ clustering coef	0.245|0.031(1)	0.241|0.0226(1)	0.243|0.0248(1)	0.247|0.029(1)	0.245|0.0303(1)	0.2205
$\uparrow$ modularity	0.445|0.373(0.99)	0.437|0.309(1)	0.435|0.241(1)	0.44|0.307(1)	0.445|0.376(0.99)	0.3914
n_communities	4|5.23(0)	5|5.44(0.26)	3|3.55(0.36)	4|6.16(0.03)	4|5.74(0)	2.0000
VIdist_to_GT	1.2|3.18(0)	1.34|3(0)	0.747|2.23(0)	1.17|3.1(0)	1.2|3.27(0)	0.0000

Table 2. Values of scoring functions for the karate club graph (left), compared to those of their averages for 100 randomized samples (right), and its percentile rank (parentheses).

	Louvain	leading eigen	label prop	Walktrap	spin-glass
↑ internal density	0.657|0.579(1)	0.587|0.548(1)	0.52|0.52(0)	0.586|0.408(1)	0.628|0.573(1)
↑ edges inside	186|117(0.94)	434|333(1)	1562|1562(0)	434|687(0.45)	253|149(1)
↑ av degree	14.4|11(0.96)	22.3|18.3(1)	40|40(0)	22.2|22(0.51)	17|12.5(1)
↑ FOMD	0|0(0)	0|0(0)	0.5|0.5(0)	0|0.135(0)	0|0(0)
↓ expansion	12.8|14.5(0.04)	8.89|10.9(0)	-|-(-)	8.9|12.3(0.301)	11.5|13.8(0)
↓ cut ratio	0.232|0.251(0)	0.229|0.25(0)	-|-(-)	0.229|0.254(0)	0.229|0.25(0)
↓ conductance	0.492|0.575(0.06)	0.285|0.381(0)	-|-(-)	0.286|0.52(0.247)	0.415|0.526(0)
↓ norm cut	0.585|0.665(0.04)	0.419|0.51(0)	-|-(-)	0.419|0.622(0.205)	0.522|0.626(0)
↓ max ODF	0.643|0.727(0.03)	0.472|0.544(0.04)	-|-(-)	0.443|0.619(0.301)	0.567|0.688(0)
↓ average ODF	0.649|0.726(0.05)	0.445|0.543(0)	-|-(-)	0.445|0.617(0.301)	0.58|0.687(0)
↓ flake ODF	1|1(0)	1|1(0)	-|-(-)	1|1(0)	1|1(0)
↑ clustering coef	0.87|0.585(1)	0.863|0.562(1)	0.812|0.539(1)	0.864|0.421(1)	0.884|0.583(1)
↑ modularity	0.0576|0.0158(1)	0.0558|0.0136(1)	-1.6e-14|-3.73e-16(0)	0.0553|0.000591(1)	0.06|0.0187(1)
n_communities	4|4.21(0.06)	2|2.45(0)	1|1(0)	2|19.3(0.27)	3|3.6(0)

Table 3. Values of scoring functions for the Forex graph (left), compared to those of their averages for 100 randomized samples (right), and its percentile rank (parentheses). ”-” indicates the degenerate cases for which a given score is not defined.

	Louvain	leading eigen	label prop	Walktrap	spin-glass
↑ internal density	0.235|0.183(1)	0.204|0.0997(1)	0.0574|0.0539(1)	0.444|0.244(1)	0.249|0.185(1)
↑ edges inside	5631|3779(1)	9051|5935(1)	21692|21771(0)	7123|2088(1)	5755|3409(1)
↑ av degree	35.7|24.9(1)	39|29(1)	48.4|48.4(0)	39.4|21.1(1)	36|24.9(1)
↑ FOMD	0.38|0.31(1)	0.392|0.35(1)	0.493|0.493(0)	0.384|0.244(1)	0.378|0.302(1)
↓ expansion	6.38|11.8(0)	4.69|9.74(0)	0.00334|-(-)	4.49|13.7(0)	6.2|11.8(0)
↓ cut ratio	0.0108|0.0219(0)	0.0108|0.0272(0)	0.000558|-(-)	0.00791|0.0205(0)	0.0106|0.02(0)
↓ conductance	0.205|0.391(0)	0.154|0.271(0)	0.000702|-(-)	0.22|0.57(0)	0.202|0.412(0)
↓ norm cut	0.259|0.472(0)	0.219|0.384(0)	0.111|-(-)	0.27|0.636(0)	0.257|0.487(0)
↓ max ODF	0.229|0.509(0)	0.178|0.481(0)	0.00111|-(-)	0.277|0.7(0)	0.199|0.534(0)
↓ average ODF	0.241|0.518(0)	0.185|0.425(0)	0.00113|-(-)	0.266|0.701(0)	0.238|0.54(0)
↓ flake ODF	0.759|0.999(0)	0.605|0.999(0)	0.00445|-(-)	0.727|1(0)	0.747|0.999(0)
↑ clustering coef	0.296|0.233(1)	0.152|0.221(0)	0.0437|0.217(0)	0.222|0.187(1)	0.289|0.226(1)
↑ modularity	0.373|0.149(1)	0.334|0.122(1)	0.000276|-1.13e-14(1)	0.342|0.0973(1)	0.372|0.152(1)
n_communities	14|8.1(1)	10|2.3(1)	2|1(1)	82|76.7(0.6)	16|12.4(1)

Table 4. Values of scoring functions for the News on Corporations graph (left), compared to those of their averages for 100 randomized samples (right), and its percentile rank (parentheses).

	Louvain	leading eigen	label prop	Walktrap	spin-glass
↑ internal density	109|43(1)	57.3|24.4(0.98)	158|8.93(1)	65|7.23(1)	26.2|39.5(0)
↑ edges inside	14828|10482(0.89)	11351|23434(0.33)	7350|111195(0)	10451|121642(0)	6612|4502(0.99)
↑ av degree	1047|583(1)	963|650(0.94)	902|1255(0)	1001|1322(0)	440|482(0.07)
↑ FOMD	0.44|0.26(1)	0.391|0.267(0.94)	0.386|0.488(0)	0.413|0.5(0)	0.163|0.195(0.02)
↓ expansion	113|370(0)	155|357(0)	186|36.4(1)	136|-(-)	417|420(0.35)
↓ cut ratio	0.787|2.58(0)	1|3.05(0)	1.13|17(0)	0.851|-(-)	2.66|2.55(0.91)
↓ conductance	0.113|0.414(0)	0.162|0.407(0)	0.224|0.0167(1)	0.162|-(-)	0.564|0.534(0.9)
↓ norm cut	0.133|0.471(0)	0.182|0.481(0)	0.24|0.15(1)	0.177|-(-)	0.611|0.566(1)
↓ max ODF	0.248|0.608(0)	0.303|0.569(0.0426)	0.32|0.00558(1)	0.29|-(-)	0.824|0.695(1)
↓ average ODF	0.207|0.6(0)	0.308|0.578(0)	0.353|0.0277(1)	0.296|-(-)	0.713|0.705(0.63)
↓ flake ODF	0.935|0.998(0)	0.957|0.997(0)	0.967|0.423(1)	0.962|-(-)	0.989|0.999(0)
↑ clustering coef	0.0837|0.194(0)	0.0902|0.19(0)	0.123|0.19(0)	0.112|0.191(0)	0.0351|0.195(0)
↑ modularity	0.673|0.27(1)	0.615|0.202(1)	0.615|0.1(1)	0.639|0(1)	0.226|0.251(0.07)
n_communities	10|6.22(1)	12|9.54(0.74)	31|1.94(1)	19|1(1)	12|12.8(0.27)

Table 5. Values of scoring functions for the Enron graph (left), compared to those of their averages for 100 randomized samples (right), and its percentile rank (parentheses).

<table>
<tr><th></th><th>Louvain</th><th>leading eigen</th><th>label prop</th><th>Walktrap</th><th>spin-glass</th></tr>
<tr><td>↑ internal density</td><td>45.3|3.17(1)</td><td>15.8|1.22(1)</td><td>98|1.19(1)</td><td>1.54|0(1)</td><td>4.37|5.48(0)</td></tr>
<tr><td>↑ edges inside</td><td>255578|86181(1)</td><td>1076946|223193(1)</td><td>5690431|1230459(1)</td><td>896171|0(1)</td><td>52171|113572(0)</td></tr>
<tr><td>↑ av degree</td><td>3680|342(1)</td><td>3639|499(1)</td><td>6572|1298(1)</td><td>1663|0(1)</td><td>577|337(1)</td></tr>
<tr><td>↑ FOMD</td><td>0.404|0.217(1)</td><td>0.375|0.287(0.9)</td><td>0.497|0.5(0)</td><td>0.23|0(1)</td><td>0.131|0.222(0)</td></tr>
<tr><td>↓ expansion</td><td>1594|478(1)</td><td>1615|400(1)</td><td>148|0.337(1)</td><td>2603|649(1)</td><td>3146|481(1)</td></tr>
<tr><td>↓ cut ratio</td><td>0.906|0.354(1)</td><td>1.27|0.374(1)</td><td>0.856|0.0422(1)</td><td>1.83|0.342(1)</td><td>1.81|0.401(1)</td></tr>
<tr><td>↓ conductance</td><td>0.307|0.54(0)</td><td>0.31|0.434(0.1)</td><td>0.0191|0.00021(1)</td><td>0.594|1(0)</td><td>0.858|0.49(1)</td></tr>
<tr><td>↓ norm cut</td><td>0.324|0.6(0)</td><td>0.378|0.535(0.1)</td><td>0.127|-(0.667)</td><td>0.658|1(0)</td><td>0.894|0.561(1)</td></tr>
<tr><td>↓ max ODF</td><td>0.0311|0.626(0)</td><td>0.0371|0.553(0)</td><td>0.00862|0.00189(0.9)</td><td>0.432|1(0)</td><td>0.857|0.58(1)</td></tr>
<tr><td>↓ average ODF</td><td>0.334|0.668(0)</td><td>0.478|0.563(0.1)</td><td>0.187|0.000483(1)</td><td>0.636|1(0)</td><td>0.924|0.822(1)</td></tr>
<tr><td>↓ flake ODF</td><td>0.767|0.996(0)</td><td>0.763|0.993(0)</td><td>0.209|0.0839(0.7)</td><td>0.864|0.999(0)</td><td>0.974|0.996(0)</td></tr>
<tr><td>↑ clustering coef</td><td>0.00417|0.147(0)</td><td>0.00127|0.147(0)</td><td>0.00179|0.15(0)</td><td>0.00031|0(1)</td><td>0.0038|0.143(0)</td></tr>
<tr><td>↑ modularity</td><td>0.461|0.122(1)</td><td>0.277|0.0676(1)</td><td>0.0778|0.00077(1)</td><td>0.0502|-0.00268(1)</td><td>-0.00289|0.119(0)</td></tr>
<tr><td>n_communities</td><td>26|13.7(1)</td><td>26|7.7(1)</td><td>26|3.3(1)</td><td>822|1899(0)</td><td>24|23.4(0.4)</td></tr>
</table>

Table 6. Values of scoring functions for the social network graph (left), compared to those their averages for 10 randomized samples (right), and its percentile rank (parentheses).

	original				randomized			
	VI	RMI	ARI	n_clusters	VI	RMI	ARI	n_clusters
Louvain	0.19	0.73	0.75	5.00	0.65	0.20	0.24	7.26
leading eigen	0.19	0.72	0.76	4.67	0.72	0.09	0.17	7.03
label prop	0.22	0.67	0.77	5.48	0.26	0.00	0.18	4.40
Walktrap	0.14	0.80	0.81	4.99	0.70	0.07	0.13	9.60
spinglass	0.39	0.45	0.53	5.74	0.76	0.08	0.19	7.82

Table 7. Mean values of the metrics after bootstrapping with $R = 999$, for both the WSBM graph and its randomized counterpart, for all tested clustering algorithms

	original				randomized			
	VI	RMI	ARI	n_clusters	VI	RMI	ARI	n_clusters
Louvain	0.27	0.66	0.67	5.57	0.49	0.39	0.42	5.49
leading eigen	0.28	0.64	0.72	5.66	0.55	0.30	0.39	5.79
label prop	0.26	0.63	0.71	5.01	0.50	0.36	0.33	4.86
Walktrap	0.27	0.68	0.71	5.94	0.42	0.49	0.47	6.57
spinglass	0.62	0.20	0.38	6.34	0.62	0.22	0.35	6.04

Table 8. Mean values of the metrics after bootstrapping with $R = 999$, for both the Zachary graph and its randomized counterpart, for all tested clustering algorithms

	original				randomized			
	VI	RMI	ARI	n_clusters	VI	RMI	ARI	n_clusters
Louvain	0.32	0.52	0.55	3.13	0.66	0.04	0.15	3.34
leading eigen	0.25	0.47	0.62	2.34	0.40	0.10	0.19	2.18
label prop	0.00			1.00	0.00		1.00	1.00
Walktrap	0.24	0.49	0.62	2.41	0.61	0.00	0.00	31.60
spinglass	0.28	0.54	0.60	3.07	0.63	0.08	0.18	3.10

Table 9. Mean values of the metrics after bootstrapping with $R = 999$, for both the Forex graph and its randomized counterpart, for all tested clustering algorithms

	original				randomized			
	VI	RMI	ARI	n_clusters	VI	RMI	ARI	n_clusters
Louvain	0.34	0.42	0.59	27.51	0.70	0.07	0.08	23.47
leading eigen	0.27	0.40	0.75	27.50	0.32	0.24	0.24	23.40
label prop	0.08	0.79	0.86	21.16	0.03	-0.00	0.00	16.62
Walktrap	0.30	0.42	0.50	85.00	0.18	0.06	0.07	91.70
spinglass	0.63	-0.12	0.13	30.65	0.94	-0.14	0.00	26.01

Table 10. Mean values of the metrics after bootstrapping with $R = 999$, for both the News graph and its randomized counterpart, for all tested clustering algorithms

	original				randomized			
	VI	RMI	ARI	n_clusters	VI	RMI	ARI	n_clusters_rnd
Louvain	0.30	0.60	0.54	10.34	0.63	0.09	0.12	5.48
leading eigen	0.42	0.46	0.40	12.78	0.50	0.14	0.20	5.96
label prop	0.34	0.54	0.48	16.36	0.02	0.22	0.48	1.44
Walktrap	0.32	0.58	0.51	17.08	1.36	0.00	0.03	170.93
spinglass	0.93	-0.20	0.03	12.63	0.74	0.08	0.13	9.36

Table 11. Mean values of the metrics after bootstrapping with $R = 999$, for both the Enron graph and its randomized counterpart, for all tested clustering algorithms
